# CLU Genetic Variants and Cognitive Decline among Elderly and Oldest Old

**DOI:** 10.1371/journal.pone.0079105

**Published:** 2013-11-14

**Authors:** Jonas Mengel-From, Mikael Thinggaard, Rune Lindahl-Jacobsen, Matt McGue, Kaare Christensen, Lene Christiansen

**Affiliations:** 1 The Danish Aging Research Center and The Danish Twin Registry, Epidemiology Unit, Institute of Public Health, University of Southern Denmark, Odense, Denmark; 2 Department of Clinical Genetics, Odense University Hospital, Odense, Denmark; 3 Department of Clinical Biochemistry and Pharmacology, Odense University Hospital, Odense, Denmark; 4 Department of Psychology, University of Minnesota, Minneapolis, Minnesota, United States of America; Oregon Health & Science University, United States of America

## Abstract

The *CLU* gene is one of the prime genetic candidates associated with Alzheimers disease. In the present study *CLU* genotypes and haplotypes were associated with baseline cognition and the rate of cognitive decline in two cohorts, the Danish 1905 birth cohort (93 years of age in 1998) and the Longitudinal Study of Aging Danish twins (LSADT) (73–83 year old twins in 1997). Both Mini Mental State Examination (MMSE) and a cognitive composite score was attained up to six times for up to 10 years and analysed using random effects models and vital status. The rs11136000 T allele was associated with better baseline cognitive performance both in the LSADT (effect on intercept: 0.41 95% CI [−0.04; 0.87]) and the 1905 birth cohort (effect on intercept: 0.28 95% CI [0.01; 0.55]), although it did not reach significance in the LSADT cohort. However, the rs11136000 T allele was significantly associated with a steeper decline (effect on slope: −0.06 95% CI [−0.11; −0.01]) in the LSADT cohort, but not in the 1905 birth cohort. Haplotype analyses revealed that carriers of the common rs11136000, rs1532278 and rs9331888 TTC haplotype (36%) in the *CLU* gene performed cognitively better than non-carriers in the 1905 birth cohort (effect on intercept: 0.50 95% CI [0.12; 0.91]) and carriers of a rare TCC haplotype (1%) performed worse on the cognitive composite score (effect on intercept: −1.51 95% CI [−2.92; −0.06]). The association between the TTC haplotype and better cognitive composite score was higher among those surviving past the age of 98 (p = 0.014), and among these the TTC haplotype was borderline associated with a steep decline (effect on slope: −0.13 95% CI [−0.27; 0.00]). In summery *CLU* genetic variants associate with cognition in two cohorts, but the genetic effect of *CLU* seems to regress toward the mean when aging.

## Introduction

Sustaining cognitive abilities is a fundamental element for successful aging and a major component of quality of life. The neurodegenerative Alzheimer's disease (AD) is one of the factors that undermine the general health of elderly. Early onset AD has been linked to rare mutations in genes encoding proteins or peptides in the synthesis of ß-amyloid (Aß), i.e. APP, PS1 and PS2 [1]. The much more abundant form of late onset AD is also heritable with genetic contribution of approximately 60% [2] that is caused by complex genetics of which the APOE allele ε4 is the primary genetic candidate risk factor [3]. Additionally, the APOE alleles ε4 and ε2 have been associated with cognition in the normal range even in the oldest old [4].

Genes and AD have been intensively studied adding up to more than 1200 published genetic association studies [5]. Recent advantages in multistage Genome Wide Association Studies (GWAS) of AD have led to findings of many new genetic risk factors. One of the most appealing findings came from two large independent GWAS studies where association was established with a *CLU* rs11136000 genetic variant and at the same time associations with two other variants in the genes *CR1* and *PICALM* were established [3], [6], [7]. The association between rs11136000 and AD has since been replicated in numerous populations, and a meta-analysis has estimated an odds ratio of 0.89 95%CI [0.86; 0.91] indicating that rs11136000 minor T allele is less frequent in AD patients in cross-sectional studies [3], [5]. To our awareness the rs11136000 genetic variation does not have any functionality e.g. causing amino acid change or splice-site alteration, since it is situated internally in intron 1 or 3 dependent on the different transcripts. Neither have there been suggestions that it causes alterations in regulatory elements, thus it is more likely to be in linkage disequilibrium (LD) with one or more functional causal variants. Such causal variants could be variants in the exons of *CLU* but several attempts to find common types have failed [8]–[11], while rare coding variants have proven to be associated with AD, although these do not account for the rs11136000 AD association [11]. Among the *CLU* variants in LD with rs11136000 are three variants rs9331888, rs1532278 and rs9331908 of particular interest. Bioinformatic studies have suggested that rs1532278 is located in intron 3 in a region that strongly resembles a regulatory element [11]. Also the rs9331888 risk variant was found to eliminate binding sites for NFkapB (nuclear factor kappa-light-chain-enhancer of activated B cells) and EBF (early B-cell factor) while it introduces a new binding site for HSF (heat shock factor protein 1) [12].

*CLU* genetic variants failed to interact with APOE alleles ε4 in genetic AD association studies in contrast to other genetic variants e.g. in the *PICALM* gene, despite suggestions that CLU like APOE is involved in trafficking of lipid particles [13], [14]. Also *CLU* genetic variants have failed to be significantly associated to biomarkers of AD i.e. Ab42, ptau181 [15], [16], neurofibrillary Tangles/plaque formations [17], the clusterin plasma level [18] and the general transcription of CLU [7], [19]. However, *CLU* genetic variants were associated with the mRNA isoform transcripts NM_203339 expressed specifically in the temporal lobe, of which rs9331888 was the variant most strongly associated with the transcript and it was suggested to be one of the functional DNA variants underlying the association between rs11136000 and AD [12]. Although these results may be subject to type 2 errors it is likely that the results indicate that the effect of *CLU* genetic variants were not restricted to the AD pathology and may act through an alternative mechanism e.g. age-related cognitive decline in general or effects may even be restricted to specific neurological centres or cell types. Brain imaging studies have provided evidence of association between the rs11136000 genetic variant and white matter integrity among young adults with normal cognitive abilities, but no association was observed with gray matter volume as was found for e.g. a *CR1* genetic variant [20], [21]. The *CLU* genetic variants have failed to be significantly associated with decline in memory in a longitudinal study in contrast to what was observed for a *CR1* genetic variant [17], although others have indicated that *CLU* may affect the decline in some groups [22]. Also the *CLU* rs11136000 genetic variant was not associated with brain volume in patients with multiple sclerosis [23]. Among the oldest old, we have previously shown that the *CLU* rs11136000 genetic variant was associated with cognition in a cross-sectional study and carriers of the T allele had a better cognitive performance [24]. Thus these results emphasise the influence of *CLU* genetic variants on general cognition.

In the present study we intended to scrutinize the associations between key candidate genetic variants in the *CLU* gene and both the baseline cognitive performance at 73 years of age for the participants in the Longitudinal Study of Danish Twins (LSADT) and at 93 years of age for the participants in the Danish 1905 birth cohort study. We also investigated associations of the CLU variants with cognitive decline in the two cohorts. Cognitive performance was measured by both the MMSE and a cognitive composite score. Our initial hypothesis was that alleles associated with better cognitive performance also associate with staying more cognitively intact by having a less steep decline. Furthermore we tested whether longevity would have an impact on the effect of the *CLU* gene on cognitive performance.

## Materials and Methods

### Subjects

The persons included in this study were drawn from participants of two population-based nationwide surveys conducted at the University of Southern Denmark: The Longitudinal Study of Aging Danish Twins (LSADT) [25], [26] and The Danish 1905 birth cohort Study [27]. LSADT is a cohort sequential study of Danish twins aged 70 years and older and was initiated in 1995 with follow-up every second year through 2005. In LSADT a total of 689 participants provided a blood sample at the assessment in 1997. Of the 689 participants, 573 were selected because they were 73–83 years of age and thus at least one decade younger than participants from the Danish 1905 birth cohort. The Danish 1905 birth cohort study is a prospective investigation of an entire birth cohort. The survey was initiated in 1998, when the participants were 92–93 years and followed by three follow-up studies of the participating survivors in 2000, 2003 and 2005. Of the 3,600 individuals still alive at intake, 2,262 participated, and 1,651 provided either a blood spot sample or a cheek swap at their first assessment in 1998. Each survey of the LSADT and 1905 birth cohort studies comprises multidimensional face-to-face interviews focusing on health and lifestyle issues, as well as objective assessment of cognitive and physical abilities. Written informed consent was obtained from all participants and both studies were approved by the Danish Scientific-Ethical Committees.

### Cognitive functions

Cognitive functioning was assessed using a 5-component cognitive composite score and the Mini Mental State Examination (MMSE) [28]. The widely used MMSE ranges from 0 to 30 and can be graded as severely impaired for scores between 0 and 17, mildly impaired for scores between 18 and 23 and normal for scores between 24 and 30. The 5-component cognitive composite measures were originally selected to represent tasks that are sensitive to normative age changes, which can be reliably and briefly assessed by lay interviewers. The specific tasks included a fluency task, which involved the number of animals an individual could name in a 1-minute interval, forward and backward digit span, and immediate and delayed recall of a 12-item list. The cognitive composite score was computed by taking the sum of the five standardized measures, separately from each cohort (using means and SDs from the initial LSADT assessment in 1995 and the Danish 1905 birth cohort survey assessment in 1998, respectively) and has been used in numerous publications for a decade [29].

### Survival

Each participant was followed from the baseline interview date until December 31, 2011, in the Danish Civil Registration system, which registers date of death, thus determining the age of death [30].

### Genetic analyses

We searched the literature for genetic studies including *CLU* gene variants associated or linked with AD, normal cognitive performance and neurological diseases e.g. multiple sclerosis. A total of 46 papers that included genetic variation in or proximal to the *CLU* gene were reviewed. Nine potential single nucleotide polymorphisms were found from the literature search, which was further reduced to four single nucleotide polymorphisms rs11136000, rs9331888, rs1532278 and rs9331908 and included in the present study since these were potentially functional variants or key variants in haplotypes. DNA was isolated from cheek swabs or blood spots, with the use of QIAamp DNA Mini Kit (Qiagen, Hilden, Germany) [31]. DNA from whole blood was isolated using a salting out method. Genotyping of the single nucleotide polymorphisms rs11136000, rs9331888, rs1532278 and rs9331908 were performed by allelic discrimination using pre-designed Taqman® SNP genotyping assays (Applied Biosystems). Reactions were conducted in a 10 µl volume using the conditions recommended by the manufacturer. PCR was performed in the Step One Plus™ Real-Time PCR system and genotypes called using the Step One™ Software version 2.1 (Applied Biosystems).

### Statistical analyses

A random effects model was used to perform analyses of the associations between the *CLU* gene variants and both the intercept and the slope of cognitive functioning separately within each cohort i.e. those 73–83 and 93 years of age at baseline. The intercept was defined as the level of the cognitive functioning at the age of 73 and at the age of 93 for each of the respective groups. Cognitive functioning was analyzed as continuous variables using the cognitive composite score and the MMSE. The CLU gene variants were both analysed as single genotypes and as a haplotypes. The genotype analyses were carried out using an additive model, hence the genotypes were recoded as 0, 1 and 2, where 0 were homozygotes for the major allele, 1 were heterozygotes, and 2 were homozygotes of the minor allele, while haplotype analyses were carried out using combinations of the rs11136000, rs1532278 and rs9331888 genetic variants and were coded as a binary present/not present in participants. The random effects models were only analyzed with a random intercept for each participant since random effects of the slope for each participant did not reach the 5% level of significance when genetic variants were included, indicating that everyone within each *CLU* gene variant has the same slope, but this was probably due to lack of power to estimate this random effect of the slope. A quadratic function of the cognitive decline did not have a better fit than a linear decline, since the quadratic term did not reach the 5% level of significance. Sex did not reach a 5% level of significance on cognitive measures. Since the random effects model assumes an immortal cohort which is not fulfilled, especially not in the oldest old, we also separated the 92–93 year old participants into two groups according to their longevity and performed random effects models separately on each group. High longevity was defined as death at or after the age of 98, and low longevity was defined as death before turning the 98 years of age, and thus not being able to participate in the third assessment. Post-hoc analyses were repeated between the cognitive composite score and single SNPs and haplotypes by restricting the sample to include only those cognitively nonimpaired (MMSE 24+). Corrections for multiple testing were not applied to these analyses of allele effects, since an a priori hypothesis of association was assumed for each of the selected SNP alleles and for each stratification step. Haplotypes (1df) were deduced empirically using data from the unrelated individuals from the LSADT and 1905 birth cohort in Haploview 4.1 [32]. The random effects models were performed using STATA 11.2 (StataCorp, Texas, USA).

## Results

### Cognitive decline in the 1905 birth cohort and the LSADT cohort

**Figure 1:**
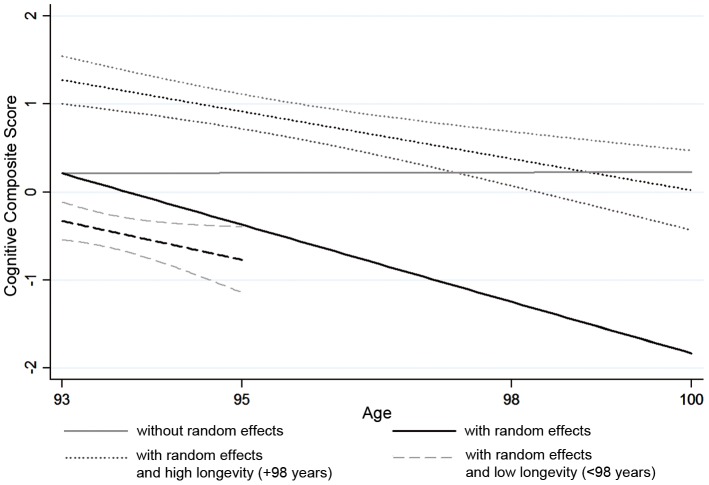
Illustration of cognitive composite score for the 1905 birth cohort stratified by participation at 93 years of age, and at follow-up assessments at 95, 98 and 100 years of age. The gray lines surrounding the lines for high and low longevity show the estimated 95% confidence intervals.

In the 1905 birth cohort, the cognitive composite score was standardised to a mean of 0.0 and a standard deviation (SD) of 1.0 at the baseline assessment at age 93 years (intercept) and was not significantly different at the later ages i.e. 95, 98 and 100 evaluated from a non-random effect model (Figure 1). Among the 1651 participants who donated blood and were included in the current analyses the mean cognitive composite score was slightly higher than the combined cohort of both blood donors and non-blood donors (table 1). Individual differences in the cognitive composite score for the complete cohort of blood donors were evaluated using a random effects model that included an intercept (mean intercept: 0.21 95%CI [0.03, 0.39]) and an annual linear rate of decline (mean slope: −0.29 95% CI [−0.35, −0.24]), which was significantly less than 0.0. The difference between the non-random effect model and the random effect model is mainly due to the high mortality among the participants with low cognitive scores. We also stratified the random effects model into those dying before the age of 98 dubbed as having low longevity and those dying at 98 year of age or later dubbed as having high longevity. For those with high longevity the baseline cognitive composite score (p<0.001) was higher and the cognitive composite score slope (p = 0.02) was less steep than for those with low longevity. In both longevity strata the annual linear rate of decline was slightly less steep than that estimated in the complete cohort of blood donors (figure 1).

**Table 1:** Descriptives in the LSADT cohort at baseline in 1997 and the 1905 birth cohort at baseline in 1998.

	LSADT cohort	1905 cohort
Age	78.8 years (73–95)	92–93 years
Number of individuals	689	1651
Mean composite cognitive score	0.95	0.23
SD composite cognitive score	3.33	3.47
Mean MMSE	25.90	21.77
SD Mean MMSE	3.68	5.78
Median MMSE	27	23
% Nonimpaired	80.7	47.3
% Mildly impaired	15.3	32.6
% Severely impaired	4.0	20.0
rs9331908 minor allele (T) frequency (%)	0.37	0.35
rs11136000 minor allele (T) frequency (%)	0.36	0.38
rs1532278 minor allele (T) frequency (%)	0.36	0.37
rs9331888 minor allele (G) frequency (%)	0.39	0.31

Like the cognitive composite score the mean MMSE at 93 years of age estimated by the random effect model (mean intercept: 21.72 95%CI [21.42; 22.01]) was similar to that estimated without introducing the random effect model (21.78 95%CI [21.51; 22.06]) and the MMSE decline (mean slope −0.76 95%CI [−0.84; −0.68]) was steeper than that estimated without the random effect i.e. differences at the later ages (−0.29 95% CI [−0.41; −0.07]).

The LSADT cohort participants (N = 573) were defined by being from 73 to 83 years of age when donating blood in 1997 and were assessed every second year from 1995 to 2007. The random effect of the baseline cognitive composite score was high (mean intercept: 2.07 95% [1.77; 2.38]) for a 73 year old participant and a significant yearly decline was observed (mean slope: −0.13 95% CI [−0.16; −0.09]). For MMSE in LSADT the mean intercept was estimated for a 73 year old participant (mean intercept: 27.26 95% CI [26.91; 27.61] and a significant yearly decline was (mean slope: −0.21 95% CI [−0.24; −0.17]) likewise observed.

### Genetic variants in the *CLU* gene and cognitive performance

Genotypes in the *CLU* gene were obtained from participants in the 1905 birth cohort for the genetic variant rs9331908 (N = 1541), rs1136000 (N = 1380), rs1532278 (N = 1518) and rs9331888 (N = 1548). From the LSADT survey, genotypes were also obtained for rs9331908 (N = 665), rs1136000 (N = 670), rs1532278 (N = 673) and rs9331888 (N = 669). All genetic variants were in Hardy-Weinberg equilibrium. The allele frequencies in 1997 in the LSADT cohort were not different from those in 1998 from the 1905 birth cohort as shown in table 1, and were similar to frequencies in the European descendants in the HapMap database. Also the allele frequencies were not different among those who attended the first assessment and those who attended each of the follow-up assessments, which indicated no obvious genetic selection had occurred with age (data not shown).

The rs11136000 minor T allele was non-significantly associated (p = 0.08) with better baseline level of cognitive performance (effect on intercept: 0.41 95% CI [−0.04; 0.87]) in LSADT at 73 years of age with higher effect than in the 1905 birth cohort at 93 years of age (effect on intercept: 0.28 95% CI [0.01; 0.55]) which did reach significance (p = 0.047) (table 2). The minor T allele of rs11136000 was, however, also significantly associated (p = 0.009) with a steeper yearly decline (effect on slope: −0.06 95% CI [−0.11; −0.01]) in the cognitive composite score, but no such association (p = 0.95) with decline was observed in the 1905 birth cohort (effect on slope: 0.002 95% CI [−0.08; 0.08]). Consistency in direction of association was shown for both the minor alleles of rs1532278 and rs11136000 as expected, since they are in almost perfect linkage disequilibrium (D′ = 0.99, r^2^ = 0.95). Also association between the minor alleles of rs1532278 and rs11136000 and baseline level of MMSE was consistent in both cohorts, but no associations reached a 5% significance level (table 2).

**Table 2:** *CLU* genotype association with cognitive composite score and MMSE by random effect models at intercept of 73 years of age and yearly decline (slope) for participants in the LSADT cohort (73–83 years of age in 1997) and at intercept of 93 years of age and yearly decline (slope) for participants in the 1905 birth cohort.

		cognitive composite score				MMSE			
	No. ind.	Effect on intercept [95%CI]	p-value	Effect on slope [95%CI]	p-value	Effect on intercept [95%CI]	p-value	Effect on slope [95%CI]	p-value
**LSADT-cohort**									
rs9331908	541/	−0.27	0.26	0.06	0.006	0.05	0.85	0.01	0.96
	542	[−0.72; 0.19]		[0.02; 0.11]		[−0.47; 0.56]		[−0.05; 0.06]	
rs11136000	544/	0.41	0.08	−0.06	0.009	0.12	0.65	0.03	0.27
	545	[−0.04; 0.87]		[−0.11; −0.01]		[−0.40; 0.64]		[−0.02; 0.09]	
rs1532278	547/	0.43	0.07	−0.05	0.02	0.09	0.73	0.04	0.12
	548	[−0.03; 0.89]		[−0.10; −0.01]		[−0.43; 0.61]		[−0.01; 0.10]	
rs9331888	545/	−0.34	0.17	0.06	0.01	0.03	0.90	0.01	0.83
	546	[−0.81; 0.14]		[0.02; 0.11]		[−0.50; 0.57]		[−0.05; 0.06]	
**1905 birth cohort**									
rs9331908	1478/	−0.18	0.18	−0.002	0.96	−0.05	0.83	0.01	0.82
	1479	[−0.45; 0.55]		[−0.08; 0.08]		[−0.50; 0.40]		[−0.11; 0.14]	
rs11136000	1328/	0.28	0.047	0.002]	0.95	0.24	0.29	0.05	0.42
	1329	[0.01; 0.55]		[−0.08; 0.08]		[−0.21; 0.70]		[−0.07; 0.18]	
rs1532278	1456/	0.21	0.12	0.007	0.87	0.08	0.74	0.05	0.41
	1457	[−0.05; 0.48]		[−0.07; 0.08]		[−0.37; 0.52]		[−0.07; 0.18]	
rs9331888	1485/	−0.31	0.03	−0.02	0.65	−0.36	0.13	−0.04	0.55
	1486	[−0.59; −0.03]		[−0.10; 0.06]		[−0.82; 0.11]		[−0.17; 0.09]	

More participants completed the MMSE than the cognitive composite score.

The rs9331888 genetic variant's minor G allele was non-significantly associated (p = 0.17) with poorer initial cognitive composite score in LSADT at 73 years of age (effect on intercept: −0.34 [−0.81; 0.14]) with similar effect in the 1905 birth cohort (effect on intercept: −0.31 [−0.59; −0.03]) which reached significance (p = 0.03). It was, however, also significantly associated (p = 0.01) with a slower rate of cognitive decline in LSADT (effect on slope: 0.06 95% CI [0.02; 0.11]), but not in the 1905 birth cohort (p = 0.65). No significant associations with initial level of MMSE or rate of decline in MMSE were observed in either of the two cohorts (table 2). These results indicate that the rs9331888 variant may also be relevant for the association between the *CLU* gene variants and cognitive composite score and the almost complete linkage disequilibrium between rs9331888 and rs11136000 (D′ = 0.99, r^2^ = 0.27) suggesting a possible additional third common haplotype.

Haplotype analyses using the rs11136000, rs1532278 and rs9331888 genetic variants indicate that carriers of the common TTC allele (36%) tended to have a better baseline cognitive composite score (effect on intercept: 0.28 95% CI [−0.35; 0.93]) in LSADT than non-carriers although it does not reach significance (p = 0.37). Similar direction of association reached significance (p = 0.01) in the 1905 birth cohort (effect on intercept: 0.51 [0.10; 0.92]). For carriers of a low frequent haplotype TCC (1%) significant association (p = 0.01) with lower initial cognitive composite score was observed in the 1905 birth cohort (effect on intercept: −1.51 95% CI [−2.92; −0.06]), but not in the LSADT cohort (effect on intercept: −0.06 95% CI [−2.91; 2.79], p = 0.97). As for genotypes, the associations between the haplotypes and baseline MMSE or decline in MMSE did not reach a 5% significance level in either of the two cohorts (table 3).

**Table 3:** *CLU* rs11136000, rs1532278 and rs9331888 genetic variants haplotype association with cognitive composite score and MMSE by random effect models at intercept at 73 years of age and yearly decline (slope) for participants in LSADT (73–83 years of age in 1997) and at intercept at 93 years of age and yearly decline (slope) for participants in the 1905 birth cohort.

		cognitive composite score				MMSE			
	Frequencies	Effect on intercept [95%CI]	p-value	Effect on slope [95%CI]	p-value	Effect on intercept [95%CI]	p-value	Effect on slope [95%CI]	p-value
**LSADT-cohort (N = 533/534) 73–83 years of age**									
H1: TTC	0.36	0.28	0.37	−0.04	0.26	−0.06	0.86	0.07	0.06
		[−0.35; 0.93]		[−0.10; 0.03]		[−0.78; 0.65]		[−0.00; 0.15]	
H2: CCC	0.32	−0.26	0.42	−0.001	0.96	−0.61	0.09	−0.002	0.96
		[−0.89; 0.37]		[−0.06; 0.06]		[−1.32; 0.10]		[−0.08; 0.07]	
H3: CCG	0.31	−0.33	0.32	0.08	0.02	0.09	0.80	−0.01	0.83
		[−0.97; 0.32]		[0.01; 0.15]		[−0.63; 0.82]		[−0.09; 0.07]	
H4: TCC	0.01	−0.06	0.97	−0.22	0.09	1.46	0.38	−0.40	0.01
		[−2.91; 2.79]		[−0.47; 0.03]		[−1.79; 4.71]		[−0.71; −0.09]	
**1905 birth cohort (N = 1180/1181)**									
H1: TTC	0.36	0.51	0.01	−0.08	0.24	0.30	0.39	−0.04	0.72
		[0.10; 0.92]		[−0.20; 0.05]		[−0.39; 0.98]		[−0.23; 0.16]	
H2: CCC	0.32	0.21	0.30	0.002	0.96	0.12	0.74	−0.07	0.49
		[−0.19; 0.61]		[−0.12; 0.13]		[−0.56; 0.79]		[−0.27; 0.13]	
H3: CCG	0.31	−0.26	0.21	−0.06	0.37	−0.14	0.68	0.05	0.60
		[−0.67; 0.15]		[−0.18; 0.07]		[−0.82; 0.54]		[−0.14; 0.25]	
H4: TCC	0.01	−1.51	0.041	0.04	0.82	−1.24	0.32	0.01	0.98
		[−2.96; −0.06]		[−0.33; 0.41]		[−3.66; 1.19]		[−0.61; 0.63]	

Post-hoc analyses were conducted among the nonimpaired participants to further deduce if the associations between genetic variants in the *CLU* gene and the cognitive composite score were driven by those cognitively impaired. The analyses conducted in the LSADT cohort showed no such indications since 13 of 16 test increased the association and three additional associations reached a 5% significance level despite the reduction of 1/5 of the sample by those who were most impaired (MMSE<24). Among participant from the1905 cohort half of the associations increased when reducing the sample to half by excluding those who were most impaired (MMSE<24). The p-values of three tests were marginally higher (ranging from 0.07–0.14) thus not reaching the 5% significance level, although it should be stressed that the associations were almost identical to those estimated for the complete sample of nonimpaired and impaired participants (table 2 and 3).

### Interaction between genetic variants in the *CLU* gene and longevity and cognition

Since high longevity was associated with a higher baseline cognitive performance, interaction between haplotypes and longevity was tested and found to be significant for the TTC haplotype (p = 0.014), which indicated the positive effect of TTC on the intercept of the cognitive composite score only apply for those with high longevity from the 1905 birth cohort. Among those with high longevity TTC carriers had a significantly higher cognitive composite score than non-TTC carriers (effect on intercept: 1.22 95% CI [0.54; 1.90]) and also a borderline higher rate of decline (effect on slope: −0.13 95% CI [−0.27; 0.00]). These results indicate that the association between *CLU* haplotypes and cognitive performance moderately regress toward the mean when aging, but is restricted to those who are further from termination of their life.

## Discussion

Numerous case-control studies have firmly replicated that the rs1113600 minor T allele occurs less frequently among AD patients than among controls, with an estimated meta-analytic odds ratio of 0.89 [3], [5]. In addition, the rs9331888 G allele has been found to contribute to the association between *CLU* and AD in a large study of European decent where haplotypes defined by rs2279590, rs11136000, rs9331888 showed higher OR for the CCC haplotype (1.12 95% CI [1.06; 1.20]) compared to the TTC haplotype and even higher (OR 1.22 95% [1.14; 1.29]) for the CCG Haplotype [6]. The CCG haplotype was likewise associated with increased risk of AD in populations of Asian descent [33], [34]. In the present work a similar CCG haplotype was associated with lower baseline cognition although it was formed by haplotypes of the three SNPs rs11136000, rs1532278 and rs9331888 in the CLU gene, thus substituting the non-informative rs2279590 variant with the potential functional variant rs1532278 (table 2). The new haplotypes further deduce the association between *CLU* gene variants and cognition in the normal range since the dissimilarity between the rs11136000 T allele and rs1532278 T allele situated in the common TTC haplotype (36%) was associated with better baseline cognition and the rarer TCC haplotype (1%) was associated with worse baseline cognition thus indicating that the rs1532278 T allele actually plays a significant role in the association with cognition. This finding fits rather well with a previous suggestion that the rs1532278 T allele is located in a region that resembles a regulatory element [11]. The rs9331888 G allele was in the present work associated with non-significant lower cognition and could possibly have a partial role in cognitive functioning by the suggested elimination of one binding site and generation of a novel heat shock protein binding sites [12].

Associations between *CLU* genotypes or haplotypes and baseline cognitive composite score showed consistent association between the two cohorts who were discordant by age although the effect of association appears to be stronger in the younger cohort. Likewise there was general consistency between the cognitive composite score and MMSE both in the genotype and haplotype analyses, although the associations between *CLU* genetic variation and MMSE did not reach the 5% significance level (table 2 and 3). These results suggest that the cognitive composite score and MMSE may reflect the same cognitive abilities among the participants, but that the cognitive composite score is more sensitive in association studies than MMSE, similar to what we have observed previously [35].

Although we stress the consistency between the two cohorts a draw back to our approach is that the LSADT cohort included fewer participants (approximately N = 540) than the 1905 birth cohort (approximately N = 1340), which may explain why the 1905 birth cohort associations are more likely to reach significance despite the lower effect of the CLU gene variations. The solid evidence of associations between CLU genetic variants and Alzheimer Disease from metaanalysis of tens of thousands of participants [3], [5] implies that CLU is a key gene of interest. However, cohort analyses are of importance to deduce the complexity of gene effects under different conditions. Due to our sample size and multiple analyses that assume the same a priori hypothesis of association between CLU gene variants and cognition the results could be subject to type 1 errors and replication is needed. However, to our knowledge longitudinal data on a similar cognitive composite score only rarely exists, especially among the oldest old.

The two cohorts included 4% and 20% severely cognitively impaired participants for the LSADT and 1905 birth cohort at baseline (table 1), thus illustrating the participants in the later cohort are cognitive worse. Despite the cognitive differences in the two cohorts our analyses among those nonimpaired were highly similar to the complete sample including those severely or mildly impaired. This shows that the *CLU* genetic variants apply to the majority of variance within the normal cognitive area for each age group although it must be stressed that those severely demented were proxy interview if possible and not included in the analyses. At follow-up, those who became cognitive impaired or demented were less likely to participate. The advantage of the random effect model, however, is that it estimates the individual decline of the participants taking into account the loss to follow-up, which the non-random effect model does not.

In addition to the association between *CLU* genetic variation and baseline cognitive performance we also studied the association with cognitive decline. Initially we hypothesised that association with a higher baseline level of cognition may also associate with a reduced rate of decline, such an association was found for high longevity, with consistency between cognitive composite score and MMSE (figure 1). Similar patterns of association have been described in detail elsewhere for participation in several assessments as an alternative to studying longevity [36]. However, no such tendencies were observed between *CLU* genetic variations and cognitive decline in the present study. In contrast, we observed an inverse association that indicates regression towards the mean for *CLU* genotypes and the TTC haplotype among those with high longevity. These results fit with the fact that at baseline the association between *CLU* genotypes and cognition was highest among the young elderly. The present work adds evidence on the association between *CLU* variants and cognitive decline, which has shown inconsistent results in the previous literature. Investigating non-demented participants no indication of association with cognitive decline was reported in an earlier study of two cohorts with up to 7.8 years of follow-up [17]. Likewise no *CLU* associations were reported among subjects with mild cognitive impairments [37], whereas a nominal association with cognitive decline was reported among AD patients [38]. However, evidence that has been reported using a nonlinear Bayesian trajectory approach, show that the rs11136000 risk allele for decline was the opposite of the AD risk allele, similar to the tendencies reported in the present work [39]. An alternative explanation for the effect could be that individuals with a high initial cognitive level have more room for declining compared to those with a lower initial cognitive level who have so to speak, hit the floor, thus not being able to decline to the same extend. This hypothesis could also explain why the association with cognitive decline was observed among the cognitive better functioning LSADT participants but not among the more poorly cognitively functioning 1905 birth cohort participants (table 2). Our observations do show similarity to our previous analyses of APOE and the cognitive composite score that indicated that the ε4 allele may be associated with lower cognitive performance and the ε2 has a protective effect on cognition, which may be most explicit at the most extreme ages [4]. However, the association in the present study is in itself stronger than that of the APOE alleles within the same 1905 birth cohort and thus less likely to be subject of Type 1 errors than that of the APOE alleles. This evidence could possibly also indicate that the *CLU* gene variant is associated with later onset of cognitive decline rather than having a protective role for cognitive decline as such. Alternatively, the functionality may be different between CLU and APOE, e.g. APOE alleles are associated with mortality whereas the *CLU* genetic variants associate with cognitive performance among those who are furthest from dying rather than associated with mortality itself. Such a heterogenetic effects for *CLU* genetic variants has been reported previously by Thambisetty M and co-workers although they reported the faster rates of decline in memory among carriers of the CLU rs11136000 risk allele restricted to those who became mildly cognitive impaired [22].

CLU, which is also known as APOJ or Clusterine, does share several common functionalities with APOE i.e. both CLU and APOE proteins are present in neuritic plaque and bind to amyloid beta peptides, thus stabilising them and mediating their clearance from brain. Also both proteins are included in lipid particle traffic and thus regulate cholesterol traffic [40]. Given the nature of the haplotypes of *CLU* these may be restricted to cognitive function in contrast to e.g. the APOE missense mutations like the ε2 and ε4 defined haplotypes that have multiple functionalities. This is supported by association between the *CLU* genetics and brain function in regions that play a role in memory from structural brain imaging approaches, i.e. hypothalamus [22]. Also in a proximal brain region, the temporal lobe, a specific mRNA isoform transcripts NM_203339 was positively expressed and associated with the *CLU* rs9331888 variant [41], thus the *CLU* variants seem to play a key role in the central brain. CLU is also upregulated in AD patients, mostly in astrocytes brain cells the major source of secreted Clusterine although CLU is a versatile chaperone molecule that can target multiple stress induced proteins and prevent dysfunction in multiple organs [40]. The CLU product can have either a soluble or a nuclear form determined by different transcription sites or alternative splicing. These different isoforms are induced by different factors in different cell types e.g. TGFbeta induce the nuclear form in epithelial cells, while androgen induces different CLU isoforms in prostate cells [12]. CLU is also an inhibitor of compliment activation and it has been suggested that it acts to prevent the inflammatory response associated with complement activation downstream of protein aggregation [42]. Of more general functions CLU has been suggested to play a role in apotosis, sperm maturation, endocrine secretion and membrane protections [41].

These results suggest that CLU is a multifunctional molecule and emphasises that genetic variation in regulatory elements is probably of importance for expression of different CLU transcripts and their functionality in specific organs. Also it supports the evidence that rs1532278 and rs9331888 in combination with information from rs11136000 is likely to be functionally relevant primarily in the central brain, thus affecting the cognitive performances among elderly, but as the present work suggests less at the most extreme ages.
